# Genome-Wide association between *EYA1* and Aspirin-induced peptic ulceration

**DOI:** 10.1016/j.ebiom.2021.103728

**Published:** 2021-12-02

**Authors:** Stephane Bourgeois, Daniel F. Carr, Crispin O. Musumba, Alexander Penrose, Celestine Esume, Andrew P. Morris, Andrea L. Jorgensen, J. Eunice Zhang, D. Mark Pritchard, Panos Deloukas, Munir Pirmohamed

**Affiliations:** a William Harvey Research Institute, Barts and The London School of Medicine and Dentistry, Queen Mary University of London, London UK; b Department of Pharmacology and Therapeutics, Centre for Musculoskeletal Research, University of Manchester, Manchester, UK; c Department of Health Data Science, Centre for Musculoskeletal Research, University of Manchester, Manchester, UK; d Centre for Genetics and Genomics Versus Arthritis, Centre for Musculoskeletal Research, University of Manchester, Manchester, UK; e Department of Cellular and Molecular Physiology, University of Liverpool, Liverpool, UK

**Keywords:** NSAID, ulcer, Aspirin, GWAS

## Abstract

**Background:**

Low-dose aspirin can cause gastric and duodenal ulceration, hereafter called peptic ulcer disease (PUD). Predisposition is thought to be related to clinical and genetic factors; our aim was to identify genetic risk factors associated with aspirin-induced PUD.

**Methods:**

Patients (n=1478) were recruited from 15 UK hospitals. Cases (n=505) were defined as patients with endoscopically confirmed PUD within 2 weeks of using aspirin and non-aspirin Non-Steroidal Anti-Inflammatory Drugs (NSAIDs). They were compared to two control groups: patients with endoscopically confirmed PUD without any history of NSAID use within 3 months of diagnosis (n=495), and patients with no PUD on endoscopy (n=478). A genome-wide association study (GWAS) of aspirin-induced cases (n=247) was compared to 476 controls. The results were validated by replication in another 84 cases and 162 controls.

**Findings:**

The GWAS identified one variant, rs12678747 (*p*=1·65×10^−7^) located in the last intron of *EYA1* on chromosome 8. The association was replicated in another sample of 84 PUD patients receiving aspirin (*p*=0·002). Meta-analysis of discovery and replication cohort data for rs12678747, yielded a genome-wide significant association (*p*=3·12×10^−11^; OR=2·03; 95% CI 1·65-2·50). Expression of *EYA1* was lower at the gastric ulcer edge when compared with the antrum.

**Interpretation:**

Genetic variation in an intron of the *EYA1* gene increases the risk of endoscopically confirmed aspirin-induced PUD. Reduced *EYA1* expression in the upper gastrointestinal epithelium may modulate risk, but the functional basis of this association will need mechanistic evaluation.

**Funding:**

Department of Health Chair in Pharmacogenetics, MRC Centre for Drug Safety Science and the Barts Cardiovascular NIHR Biomedical Research Centre, British Heart Foundation (BHF)

**Research in context**

**Evidence before this study**

Aspirin and non-aspirin NSAIDs are amongst the commonest causes of peptic ulcer disease, affecting either the stomach and/or duodenum

Inhibition of cyclo-oxygenase 1 by NSAIDs reducing mucosal protection to gastric acid by prostaglandins is the most widely accepted mechanism for the tissue injury

The mechanism of NSAID-induced peptic ulceration is complex, and multiple interacting pathways in addition to mucosal protection are involved.

Genetic predisposing factors have been postulated and have focused on genes encoding cytochrome P450 and the cyclo-oxygenase enzymes, with contradictory findings.

**Added value of this study**

We performed a genome-wide association study that identified an intronic variant in the *EYA1* gene associated with endoscopically confirmed aspirin-induced peptic ulceration

Replication was shown in another cohort with aspirin-induced peptic ulceration, but the locus was not associated with ulceration associated with non-aspirin NSAIDs.

RNA sequencing of gastric biopsy samples from patients with bleeding peptic ulcers showed *EYA1* expression at the ulcer edge was lower than in the antrum.

**Implications**

We provide evidence that *EYA1* is a novel locus that predisposes to endoscopically confirmed aspirin-induced peptic ulceration. This may provide a potential pharmacogenetic biomarker and may serve as a target for future preventive anti-ulcer therapies.

Alt-text: Unlabelled box

## 1 Introduction

Low-dose aspirin (75-325mg/day) is taken by up to 30% of the general population [1], its use rising with age. Approximately 30 million people in the US take non-aspirin non-steroidal anti-inflammatory drug (NSAIDs) every day [2]. Up to 25% of all reported adverse drug reactions (ADRs) can be attributed to NSAIDs [3]. Upper gastrointestinal (GI) or peptic ulceration is one of the commonest ADRs: a population-based UK cohort study showed an incidence of NSAID-induced symptomatic, uncomplicated peptic ulcer disease of 1·03 cases per 1000-person years, with a relative risk of 2·9 (CI 2·3-3·6) for aspirin and 4·0 (CI 3·2-5·1) for non-aspirin NSAID users, compared to non-users [4]. Low-dose aspirin also increases the risk of major GI bleeding [5], especially in those over the age of 75 years [6]. Our epidemiological study of 18,820 patients showed that NSAIDs (including aspirin) were the commonest cause of ADR-related hospital admission, often due to upper GI ulceration and its complications [7]. It has been estimated that annually between 5000-16500 deaths in the USA and between 400-1000 deaths in the UK are directly attributable to NSAID-induced upper GI ulceration and GI hemorrhage [8], [9], [10].

Genetic factors may play a role in predisposing to NSAID-induced peptic ulcer disease (PUD) [11]. Many NSAIDs are metabolised by cytochrome P450 2C9 (CYP2C9), but candidate-gene studies of *CYP2C9* polymorphisms have provided conflicting evidence, with some reporting that low-activity *CYP2C9* gene variants predispose to NSAID GI complications [12,13], while others found no association [14]. Indeed a meta-analysis suggested that CYP2C9*3 but not *2 was a strong predictor of NSAID-induced ulcer and bleeding risk [15]. We evaluated the whole *CYP2C* gene cluster on chromosome 10 using the cohort reported herein, and found an association with a CYP2C19*17 gain of function polymorphism [16]. This was postulated to affect the metabolism of arachidonic acid, which is known to play a role in PUD.[17] Studies of pharmacodynamic genes such as cyclooxygenase 1 and 2 (*COX-1 and COX-2*), the pharmacological targets of most NSAIDs, again have produced contradictory findings [18], [19], [20].

Given that there are few consistent, reproducible data supporting the role of pharmacokinetic or pharmacodynamic gene polymorphisms in the pathogenesis of NSAID-induced GI complications, we have undertaken a genome wide association study (GWAS) to identify, using a “hypothesis-free” approach, common novel genetic loci as risk factors for endoscopically confirmed aspirin-induced PUD. We focused on aspirin because this was the most common NSAID associated with peptic ulceration in our cohort.

## 2 Methods

### 2.1 Patients and outcomes

Our study was designed to identify and recruit patients with NSAID-induced (including low-dose aspirin) PUD. Patient recruitment for the study has been previously described [16]. Briefly, patients who had undergone endoscopy for suspected PUD between July 2005 and June 2011 were identified from endoscopy databases at 15 hospitals in the United Kingdom and invited by telephone or email to take part in the study. Patients were also recruited prospectively (from January 2008 onwards) as hospital inpatients or when attending for endoscopy.

The final study cohort consisted of 1478 patients, who fell into 3 distinct phenotypes: cases were defined as patients with endoscopically confirmed PUD within 2 weeks of using NSAIDs (n=505). PUD was defined either from the endoscopy reports as a mucosal break ≥3 mm in diameter, or from the description of the endoscopist if size was not specified. These were compared to two control groups:

• patients with endoscopically confirmed PUD who did not have any history of NSAID use within 3 months of diagnosis (control group A; n=495); and
• patients with no PUD on endoscopy, some of whom were taking NSAID (control group B; n=478).

### 2.2 Ethics

Approval for the study, which conforms to the Declaration of Helsinki, came from the Liverpool (Adults) Research Ethics Committee (reference number 07/H1005/119) and informed consent was obtained from all eligible patients.

### 2.3 Genotyping, data calling and QC

Of the 1478 patients recruited, DNA from 723 patients (PUD cases, n=247 (all aspirin-induced); control group A, n=245; control group B, n=231) were assayed on the Illumina Omni 2·5 single nucleotide polymorphism (SNP) array (Illumina, San Diego, CA, USA); bead chips were scanned with an iScan. Intensity data, normalized according to the standard Illumina algorithm, was extracted and genotypes called using Illuminus [21]. Sample call rate was calculated and Illuminus re-run was performed using only samples with a call rate of at least 90% to improve cluster definition.

Samples having a call rate of less than 95% or having autosomal heterozygosity values in the tail of the distribution were excluded. Chromosome X heterozygosity was used to predict gender (samples with values less than 4% are predicted as male, those with values over 15% are predicted as female); this was compared to the gender in the original documentation, and discrepancies resolved, or samples excluded. A pairwise comparison was run for all samples using 400 independent common SNPs to identify duplicate samples. Genotypes for each sample were compared to the molecular fingerprint – a set of 26 markers typed using the MassArray iPLEX platform (Agena Bioscience Inc., San Diego, CA, USA)- to eliminate the possibility of arraying errors.

A principal component analysis (PCA) was performed together with Hapmap 3 [22] samples in order to identify non-European ancestry outliers. Identity by descent (IBD) was calculated for all pairs of samples using PLINK 1·9 [23,24], and one sample would be excluded from each pair for which pi-hat was greater than or equal to 0·25 (second degree relatives). A flowchart of the whole sample QC process is available in Supplementary Figure 1.

All variants with a call rate below 98% were excluded, and those with a minor allele frequency (MAF) below 3% were excluded if their call rate was below 99%. Variants with an exact *p* -value for deviation from Hardy-Weinberg equilibrium of below 10^−4^ were also excluded (Supplementary Figure 2).

### 2.4 Imputation

Prior to phasing using SHAPEIT2 [25], variants whose MAF was below 1% were excluded; the imputation was carried out with IMPUTE2 [26], using the multi-ethnic 1000 Genomes Project Phase 3 [27] integrated variant set release in NCBI build 37 as the reference panel. Post-imputation, variants with an info score below 0·8 were excluded, as well as variants with a non-unique genomic location.

### 2.5 Statistics

To maximise power for discovery of loci associated with NSAID-induced PUD, we considered all controls, irrespective of NSAID use. Consequently, by design, NSAID use was fully confounded with outcome since all cases were NSAID users. Potential confounders (demographic and clinical) tested univariately for association with the outcome were: age, gender, smoking status (three categories: non-smoker, ex-smoker, current smoker), alcohol consumption (AUDIT[28]), calcium supplements (binary), selective serotonin reuptake inhibitor (SSRI) (binary), steroids (binary), anti-coagulants (binary), anti-platelet agents (binary), anti-secretory drugs (binary), proton pump inhibitors (ppi) (binary), *Helicobacter pylori* (*H. pylori*) status (binary), history of *H. pylori* (binary), history of gastric disease (binary), history of duodenal disease (binary), history of gastro-intestinal disease (binary), history of cardiovascular disease (binary), history of respiratory disease (binary), history of renal disease (binary), history of neurological disease (binary), history of diabetes mellitus (binary), history of hepatic disease (binary), and history of muscular problems (binary). History of cardiovascular disease, history of diabetes mellitus, and history of respiratory disease were excluded because of their likely confounding with NSAID use, and hence outcome, by design.

To avoid collinearity, all potential confounders were checked for pairwise correlation using Pearson's correlation test in R. The aforementioned confounders were included in a stepwise variable selection in R [29] to determine the variables to be used as covariates in association analyses. Association testing of each SNP was undertaken in a logistic regression framework, under an additive dosage model in the minor allele, using SNPTEST [30], with a MAF cut-off of five percent, and genomic control inflation was calculated. Our study design allowed for the possibility that we would identify variants associated with NSAID use in the general population, and so we also tested for association of identified loci with NSAID use amongst controls only and excluded from replication any that demonstrated nominal significance (p<0·05). The Manhattan plot was prepared using an in-house Python script. Regional visualisations of the Manhattan plot were produced using LocusZoom software [31].

### 2.6 Replication cohort

From the total number of patients recruited, 515 self-reported white European patients with endoscopically confirmed PUD (206 cases) and 309 controls (124 control group A, 185 control group B) were included in the replication cohort. Cases and controls included in the replication cohort were recruited later than those included in the discovery cohort. Lead variants with a p-value below 5*10^−6^ in a clear delineated LD block in the association with aspirin-induced PUD in the discovery cohort were subsequently typed in the replication cohort using the Agena MassArray iPLEX platform (Agena Bioscience Inc, San Diego, CA, USA) according to the manufacturer's protocols and subsequently tested in using the same logistic regression model and methodology in SNPTEST as previously described. SNPs were excluded when MAF<0·01, call rate <95% and HWE *p*>0·0001 [exact test]. Samples were excluded if genotyping call rate <90%. Meta-analysis combining the association summary data of both cohorts was undertaken under an inverse-variance weighted fixed-effects model using GWAMA [32].

### 2.7 RNA sequencing of gastric biopsies

A subset of 10 PUD patients (3 aspirin only, 4 aspirin plus other NSAID, 3 non-aspirin NSAID) from the discovery cohort were included in the biopsy cohort. Biopsies were taken from both antrum and ulcer edge and were typically 2-3mm in size. Total RNA extraction, from a single biopsy sample was undertaken, using the miRNeasy® mini kit (QIAGEN Sciences, Germantown, MD, USA) according to the manufacturers protocol. RNA integrity number (RIN) was determined using the Agilent 2100 Bioanalyser with the RNA Nano 6000 kit according to the manufacturers protocol (Agilent Technologies, Santa Clara, CA, USA). A RIN cut-off of 7 was applied to the samples. RNA samples were poly-A selected using Invitrogen Dynabeads (Thermo Fisher Scientific, Waltham, MA, USA) and RNA-seq libraries were prepared from 50ng poly-A RNA using the Epicentre ScriptSeq v2 RNA-Seq library preparation kit according the manufacturers protocol (Illumina Inc., San Diego, CA, USA) Following 10 cycles of amplification, libraries were purified using Ampure XP beads (Beckman Coulter, Pasadena, CA, USA) and quantified using the Invitrogen™ Qubit™ fluorometer (Thermo Fisher Scientific, Waltham, MA, USA) with size distribution assessed using the Agilent 2100 Bioanalyser. Amplified libraries were multiplexed as 5 individual pools, each with 6 libraries per pool. Sequencing of the pooled libraries was undertaken using the HiSeq platform (Illumina Inc., San Diego, CA, USA). Transcript sequences were mapped to human genome 19 (hg19) reference sequence using TopHat 2·0·8 [33] and bowtie 2·1·0 [34]. Paired end mapping was applied to trimmed data and counts were reported at gene level.

### 2.8 Role of funders

Funders were not involved in the conception, undertaking, or interpretation of the research findings.

## 3 Results

Of the 723 samples genotyped on the Illumina Omni 2·5 SNP array, eight samples were excluded from all further analysis following a decision to withdraw from the study, one patient was found to have previously undergone GI surgery, and one had previous NSAID treatment, 26 samples did not cluster with the remainder of the cohort in the PCA (non-European ancestry outliers), one had an “other” self-declared ethnicity, and 10 samples had a missing phenotype, leaving 676 samples for analysis, 235 of which were cases (Table 1). All cases were taking aspirin, with eight also concomitantly taking another NSAID (Table 1).

**Table 1:** Summary of the discovery and replication cohorts included in the final genetic analysis.

	**Discovery Cohort (n)**	**Replication Cohort (n)**
**PUD ± NSAID**		
Aspirin only	226	30
Non-aspirin	0	114
Both	8	54
***Site of PUD***		
Gastric	117	107
Duodenal*	87	60
Gastroduodenal	18	18
Pyloric*	3	3
Not specified	10	11
**Total Cases**	**235**	**198**
**PUD no NSAID (Control A)**	**224**	**84**
***Site of Ulcer***		
Gastric	102	44
Duodenal	91	26
Gastroduodenal	11	3
Pyloric	7	1
Oesophageal	1	0
Not Specified	12	10
**No PUD ±/- NSAID (Control B)**	**217**	**78**
Aspirin Only	98	1
Non-Aspirin	0	8
Both	11	1
No NSAID	108	68
**Total Controls**	**441**	**162**

⁎ 1 PUD case classified as duodenal and pyloric ulcer

A total of 1,524,956 variants were used for imputation, resulting in a set of 82,310,144 variants post-imputation, of which 5,548,084 (MAF>5%) were retained, after QC, for association analysis (Supplementary Figure 2).

Univariate logistic regression analysis of clinical and demographic variables was undertaken (Table 2). History of hepatic disease, history of renal disease, history of neurological disease, history of gastro-intestinal disease, history of duodenal disease, gender, age, antiplatelet agents, smoking status, H. pylori status and steroids all remained in the final stepwise model. These 11 variables were used as covariates in the logistic regression between genetic variants and the outcome.

**Table 2:** Univariate logistic regression analysis of clinical and demographic covariates

**Variable**	**Controls (n=441)**	**Missing data (n)**	**Cases (n=235)**	**Missing data (n)**	**p-value**
*Age (years) (Mean (SD))	61·5 (14·74)	1	70·2 (10·4)	2	**2·16×10^−13^**
*Gender (Male)	213 (48·3%*)	0	76 (32·3%)	0	**7·26×10^−05^**
*Smoking Status *Non-smoker* *Ex-smoker* *Current-smoker*	185 (41·9%) 132 (30·0%) 124 (28·1%)	0	67 (28·5%) 110 (46·8%) 58 (24·7%)	0	**1·47×10^−05^**
AUDIT (Mean (SD))	4·3 (5·9)	1	3·8 (4·9)	4	0·289
*H·pylori positive	167 (41·4%)	38	134 (57·3%)	1	**1·24×10^−04^**
					
**Concomitant medications**					
*Antiplatelet agents	14 (3·2%)	0	22(9·4%)	0	**1·12×10^−03^**
*Antisecretory drugs*	12 (2·7%)	0	9 (3·8%)	0	0·431
*Anticoagulants*	24 (5·4%)	0	28 (7·7%)	0	0·258
*Calcium*	47 (10·7)	0	33 (14·0)	0	0·196
*Proton Pump Inhibitors*	188 (42·6%)	0	86 (36·6%)	0	0·128
*Selective Serotonin reuptake inhibitors*	37 (8·4%)	0	15 (6·4%)	0	0·352
*Steroids	17 (3·9%)	0	6 (2·6%)	0	0·377
					
**Previous History**					
Cardiovascular Disease	221 (50·2%)	1	197 (83·8%)	0	**6·16×10^−16^**
Diabetes Mellitus	45 (10·4%)	7	74 (31·5%)	0	**6·21×10^−11^**
*Gastrointestinal Disease	322 (73·2%)	1	112 (47·7%)	0	**8·84×10^−11^**
*Gastric Disease*	18 (4·1%)	1	7 (3·0%)	3	0·486
*Duodenal Disease	62 (14·1%)	1	45 (19·3%)	2	0·079
*Hepatic Disease	33 (7·6%)	8	18 (7·7%)	0	0·986
*H· pylori* infection	80 (18·2%)	1	36 (15·3%)	2	0·146
Musculoskeletal Problems	183 (42·1%)	6	114 (48·5%)	0	0·110
*Neurological Disease	36 (8·2%)	3	42 (17·9%)	0	**2·65×10^−04^**
*Renal Disease	32 (7·0%)	3	36 (15·3%)	0	**1·28×10^−03^**
Respiratory Disease	105 (23·9%)	1	57 (24·3%)	0	0·910

P-values in **bold** indicate independent statistically significant associations (p<0·05). *indicates variables included in the optimal stepwise logistic regression model for GWAS analysis.

**Figure 1:**
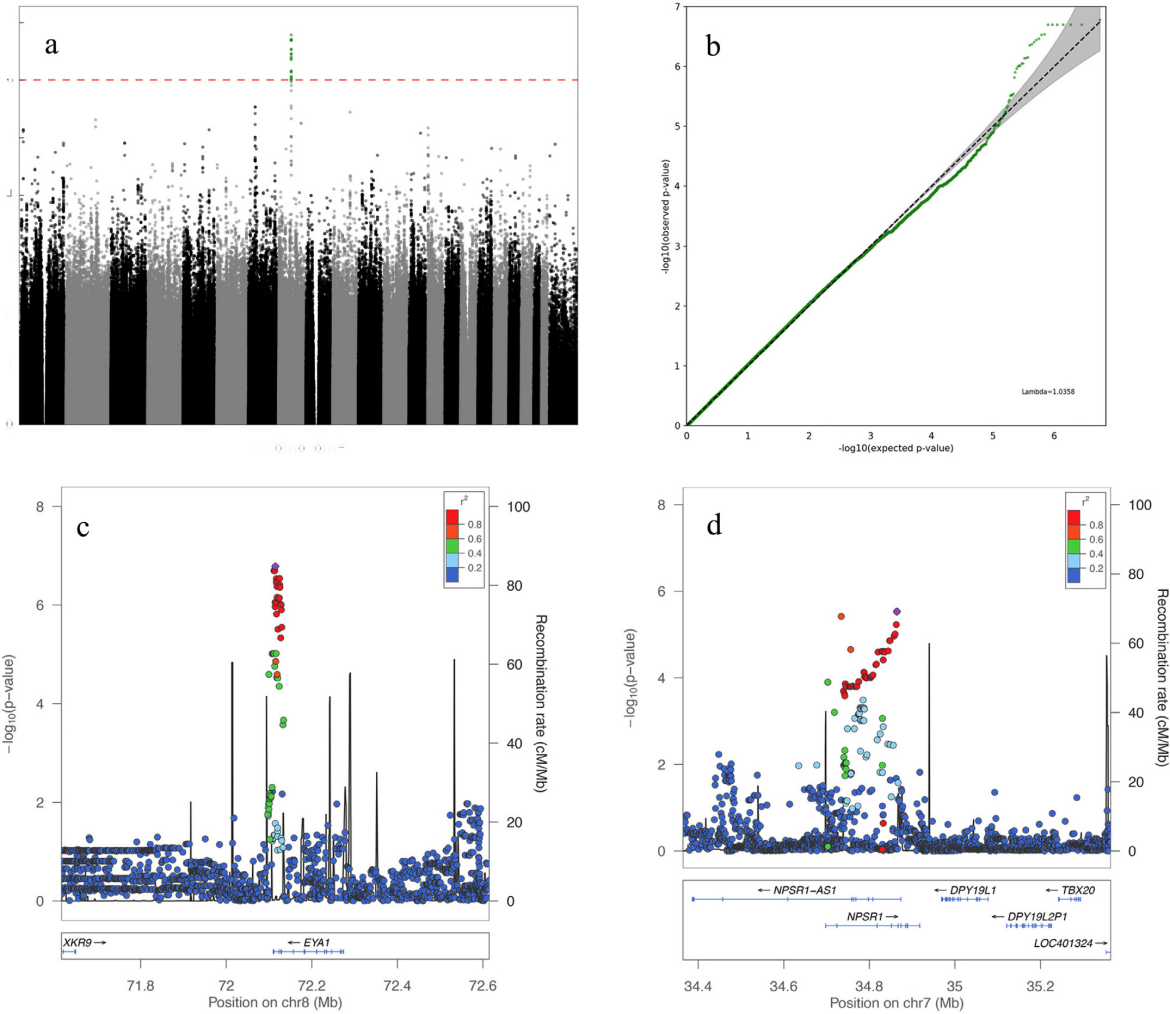
**a.** Manhattan plot of the genome wide association analysis of (aspirin)-induced peptic ulcer disease. Y-axis represents –log10 p-value for logistic regression analysis and X-axis indicates the chromosomal position of each SNP. A dotted red line marks the p=1×10^−06^ threshold, and all variants below this threshold are represented by a green dot instead of a grey/black one. **b.** Quantile-Quantile plot of the genome wide association analysis of (aspirin)-induced peptic ulcer disease. Y-axis represents –log10 p-value for logistic regression analysis and X-axis indicates –log10 of the expected p-values given the number of markers. The 95% confidence interval around expectation is represented in grey. The lambda inflation factor (1·0358) is displayed. Regional plots of rs12678747 (**c**) and rs112772601 (**d**) showing -log10(p-value) on the left Y-axis, recombination rate on the right Y-axis, and genomic location (in base pairs) on the X-axis. Pairwise linkage disequilibrium, r square, between the investigated variant (shown as a purple lozenge) and the surrounding variants is expressed using five colour coding, detailed in the top right-hand corner of each plot. Known genes are presented below the plot as arrows, indicating the location and size, as well as the direction of transcription, of each gene.

The results of the logistic regression are presented in Figure 1a and table 3. Quantiles were plotted (Figure 1b), and the inflation factor lambda was calculated at 1·0358, confirming there was no significant inflation of p-values due to population structure. After inspection of regional association plots, two loci in which the lead variant was associated at p<10^−5^ with a strong delineated signal were selected for replication. The lead variant on chromosome 8, rs12678747 (genotyped) (*p* =1·65×10^−7^ [Logistic Regression (LR)], OR=2·05, 95% CI 1·60-2·62), did not associate with NSAID use amongst controls (p=0·703 [LR]). The signal is clearly delimited and located in an intron of the gene eyes absent 1 (*EYA1*) (Figure 1c). The signal on chromosome 7, rs112772601 (*p* =2·96×10^−6^ [LR], OR=0·54, 0·39-0·75 CI), is an intronic single nucleotide deletion in the *NPSR1* gene locus, (Figure 1d). This variant did not associate with NSAID use amongst controls (*p* =0·054 [LR]). To test rs112772601 for replication we used a proxy (variant rs9655357, R^2^=0·96) which was compatible with the genotyping assay (TaqMan) used.

**Table 3:** Logistic regression results for the discovery, replication, and combined cohorts. A suitable replication protocol could not be achieved for rs112772601, and the full results for its proxy, rs9655357, are also presented here.

				**Discovery**					**Replication (aspirin cases only)**					**Combined**	
			
				**MAF**					**MAF**						
**SNP ID**	**chr**	**bp**	**AssocAllele**	**All**	**Case**	**Con**	**p-value**	**OR (95% CI)**	**All**	**Case**	**Con**	**p-value**	**OR (95% CI)**	**p-value**	**OR (95% CI)**
rs112772601	7	34,864,329	T	0·174	0.122	0.205	2·96×10^−6^	0·54 (0·39-0·75)	n/a	n/a	n/a	n/a	n/a	n/a	n/a
rs9655357	7	34,851,393	G	0·171	0.122	0.199	1·37×10^−5^	0·56 (0·40-0·77)	0·191	0.153	0.211	2·99×10^−2^	0·70 (0·41-1·10)	1·64×10^−4^	0·59 (0·45-0·77)
rs12678747	8	72,114,776	T	0·308	0.407	0.251	1·65×10^−7^	2·05 (1·60-2·62)	0·320	0.419	0.267	2·00×10^−3^	1·97 (1·34-2·92)	3·12×10^−11^	2·03 (1·65-2·50)

Replication was performed in another sample of 515 patients (replication cohort; Table 1). After accounting for missing clinical and demographic covariate data in the replication cohort, a total of 198 cases and 162 controls were included in the logistic regression analysis. Both SNPs typed in the replication cohort, rs12678747 and rs9655357, had call-rates >99·5% and conformed to Hardy-Weinberg equilibrium (*p* >0·01 [exact test]).

In our replication cohort, the *EYA1* SNP (rs12678747) showed a significant association (*p*=0·002 [LR], OR=1·97; 95% CI 1·34-2·92) when aspirin-associated cases only (n=84) were analysed in keeping with the discovery GWAS, even after Bonferroni correction. However, limiting the analysis to non-aspirin NSAIDs (n=114) or by combining all NSAIDs (n=199) did not yield a statistically significant association. Meta-analysing the discovery and replication cohort data, focusing on aspirin intake only, yielded a genome-wide significant association for rs12678747 (*p*=3·12×10^−11^ [LR]; OR=2·03; 95% CI 1·65-2·50).

The proxy for rs112772601, rs9655357, reached nominal significance (*p*=0·03 [LR]), but combined analysis with the discovery samples did not reach genome-wide significance (*p*=1·64×10^−4^ [LR]).

### 3.1 Expression of EYA1 in gastric biopsy samples

**Figure 2:**
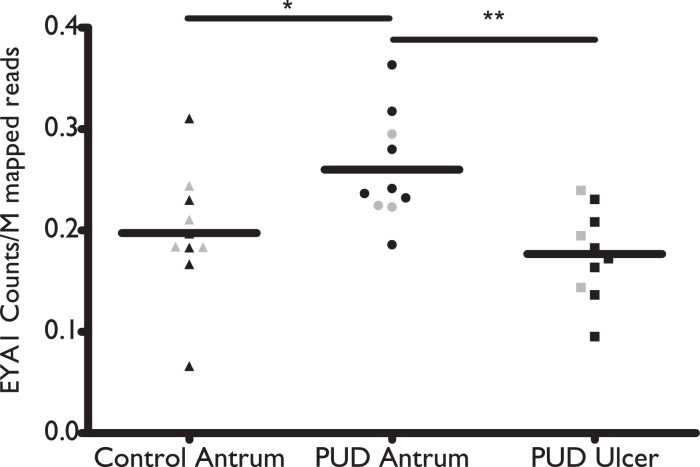
EYA1 transcript expression, determined from RNA sequencing, in antrum and ulcer biopsy tissue from aspirin-induced PUD patients (n=10). EYA counts/million mapped reads with expression data from all 10 individuals. Grey data points represent individuals who were positive for *H. pylori*. Statistically significant difference is denoted by ^⁎⁎^p<0·01 as determined by the Mann-Whitney U test

*EYAI* expression was determined by RNA sequencing in matched ulcer edge and antrum tissue samples from the 10 biopsies of NSAID-PUD patients as well as 10 healthy control antrum biopsies. Overall, expression at the ulcer edge was significantly lower than in the antrum (*p*=0·0015) [Student t-test] (Figure 2), but there was no significant difference between ulcer edge and control antrum (p>0·5).

## 4 Discussion

Evaluation of deeply-phenotyped patients with endoscopically-proven PUD and verified history of aspirin intake using a genome-wide approach has identified, for the first time, an association between common genetic variation in the EYA transcriptional co-activator and phosphatase 1 (*EYA1*) locus on chromosome 8 and aspirin-induced PUD. Although the total number of patients analysed in the discovery GWAS was modest, we were able to replicate the signal in another set of patients with aspirin-induced PUD (n=84). Our findings are consistent with the finding that pharmacogenomic predisposing loci have larger effect sizes than loci identified for complex diseases [35] which require much larger sample sizes, and rarely exceed odds ratio >2.

We did not find an association between the *EYA1* variant and non-aspirin NSAID-induced PUD, but the total number of cases was low and consisted of multiple different NSAIDs. Further work will thus be needed to determine whether the same *EYA1* variant(s) also predispose to PUD caused by non-aspirin NSAIDs (for both individual NSAIDs and as a therapeutic class). To our knowledge, this is the first GWAS focusing on aspirin-induced PUD; a previous GWAS in Japanese patients with duodenal ulceration, which did not stratify by aetiology, identified predisposing loci in the *PSCA* and *ABO* blood group genes [36], but not in *EYA1. EYA1* and *PSCA* are both on chromosome 8 but are 70Mb apart, and the underlying variants are not in linkage disequilibrium. Furthermore, no significant association with aspirin PUD was observed in our discovery cohort for either of the previously reported *PSCA* or *ABO* variants (*p*=0·14, and *p*=0·67, respectively).

*EYA1* gene mutations cause 3 genetic syndromes: branchio-oto-renal syndrome 1 (MIM 113650), branchio-otic syndrome 1 (MIM 602588) and oto-facio-cervical syndrome 1 (MIM 166780) [37], manifested by a combination of hearing loss, auricular malformations, branchial arch remnants, and renal anomalies [38]. The phenotypes of these 3 genetic syndromes are not characterised by spontaneously occurring peptic ulceration. EYA1 has a number of functions: it acts as a transcriptional activator for SIX1 [39], it is involved in organ development [40,41], DNA damage repair and cell survival [42], and angiogenesis [43]. Importantly, it also acts as a tyrosine phosphatase [39] that helps to control the apoptotic response by executing a damage-signal-dependent dephosphorylation of an H2AX carboxy-terminal tyrosine phosphate [42]. This modification determines the relative recruitment of DNA repair or pro-apoptotic factors to the tail of serine phosphorylated histone deacetylase, which in turn acts as a “decision maker” as to whether a cell undergoes cell death or repair/survival as a result of a stress signal [42]. Whether this is important in the genetic association we have identified will require further mechanistic evaluation.

Our most significant signal in *EYA1* (rs12678747, directly genotyped) is an intronic variant. Analysis using the GTex database found no significant eQTLs in stomach tissue, while initial analysis of the ENCODE database did not identify any transcription factor binding sites which are altered by rs12678747 though this does not preclude the possibility that a SNP in LD may do so. Preliminary PheWAS analysis of the *EYA1* gene locus using the GeneATLAS UK Biobank database showed that there were no statistically significant associations between *EYA1* and any relevant phenotypes. Further fine mapping and functional studies will thus be required to identify the causal variant.

A recent study utilising the UK Biobank identified loci at or near the *MUC1, MUC6, FUT2, PSCA, ABO, CDX2, GAST* and *CCKBR* genes that were associated with generalised PUD [44]. However, it is important to note that there was no stratification of patients according to aetiology (and in particular whether the PUD was due to aspirin and/or other NSAIDs). Limitations of the UKBB in relation to this phenotype are that it does not accurately record (a) the use of over-the-counter medications such as aspirin, (b) the temporal relationship between when the ulcer was diagnosed and when the patient was actually exposed to the NSAID may not be clear in all cases, and (c) the diagnosis of an ulcer varies, often self-reported, and rarely with evidence of endoscopic confirmation.

In terms of translation, we calculate that 24 individuals would need to be tested to prevent one case of aspirin-induced PUD, although this would need to be confirmed in further studies. Mechanistic knowledge of the role of EYA1 in aspirin-induced peptic ulceration may also allow the development of new agents for preventing PUD.

### 4.1 Caveats and limitations

Our study has limitations. Our overall sample size is smaller than that seen in complex disease studies as mentioned before [35], but it is compensated for by the larger effect size, careful phenotyping, and replication, though we couldn't ascertain the contribution of rarer alleles. The total number of patients for the transcriptomic analysis was low (n=10); it is however extremely difficult to recruit this patient group who are acutely ill when they attend, and in need of emergency resuscitation and endoscopy to stop bleeding. Because of the small number, we limited our analysis to ulcer and control sites only. As our study relies on a genotyping array and imputation, it is possible we haven't captured the causal allele which would require a deep sequencing-based approach.

In conclusion, we have identified that common genetic variants in the *EYA1* gene predispose to aspirin-induced peptic ulceration. Our findings do not diminish the importance of the prostanoid hypothesis in the pathogenesis of NSAID-induced peptic ulceration, but are consistent with our increasing knowledge that multiple steps are involved in the mechanism by which NSAIDs (including aspirin) induce peptic ulceration [17]. Further work is required to fully understand the mechanisms of our findings, and potential consequences for translation.

## 5 Contributors

MP, PD, DMP, COM were involved in the design and conceptualisation of the research design. ALJ, APM, SB, DFC undertook data analysis. AP, CE, DFC, and JEZ undertook sample analysis and preparation. SB, DFC, PD and MP wrote the initial draft manuscript and all authors have contributed to the writing of the submitted version. Funding for the patient recruitment was obtained by MP, while the genomics funding was obtained by both MP and PD. All authors read and approved the final version of the manuscript.

## Declaration of Competing Interest

MP has received partnership funding for the following: MRC Clinical Pharmacology Training Scheme (co-funded by MRC and Roche, UCB, Eli Lilly and Novartis); a PhD studentship jointly funded by EPSRC and Astra Zeneca; and grant funding from Vistagen Therapeutics. He has also unrestricted educational grant support for the UK Pharmacogenetics and Stratified Medicine Network from Bristol-Myers Squibb UCB. He has developed a HLA genotyping panel with MC Diagnostics, but does not benefit financially from this. None of these funding sources were used for this study. DMP reports consultancy work from Ipsen pharmaceuticals, Advanced Accelerator Applications, and Mayoly Spindler laboratories, grants from Trio Medicines Ltd, outside the submitted work. All other authors have nothing to declare.

## Data Availability

Genetic data has been deposited at the European Genome-phenome Archive (EGA, https://ega-archive.org), jointly managed by the European Bioinformatics Institute and Centre for Genomic Regulation, under accession number EGAS00001002052.

## Appendix Supplementary materials

Image, application 1

Image, application 2

Image, application 3

Image, application 4
