# Supplementary material for: Genome-Wide association between EYA1 and Aspirin-induced peptic ulceration
Source: eBioMedicine. 2021 Dec 2;74:103728. doi: 10.1016/j.ebiom.2021.103728 (PMC8646165; doi:10.1016/j.ebiom.2021.103728)
Supplement: Supplementary file 1 [file mmc1.docx]

**Caption for Supplementary Table 1:** Functional impact of variants in LD with rs12678747

**Caption for Supplementary Figure 1:** Flowchart of the sample Quality Control process. Outgoing arrows represent exclusion criteria.

**Caption for Supplementary Figure 2:** Flowchart of the variant Quality Control process. Outgoing arrows represent exclusion criteria.
