# Supplementary figures and images for: Genome-Wide association between EYA1 and Aspirin-induced peptic ulceration
Source: eBioMedicine. 2021 Dec 2;74:103728. doi: 10.1016/j.ebiom.2021.103728 (PMC8646165; doi:10.1016/j.ebiom.2021.103728)

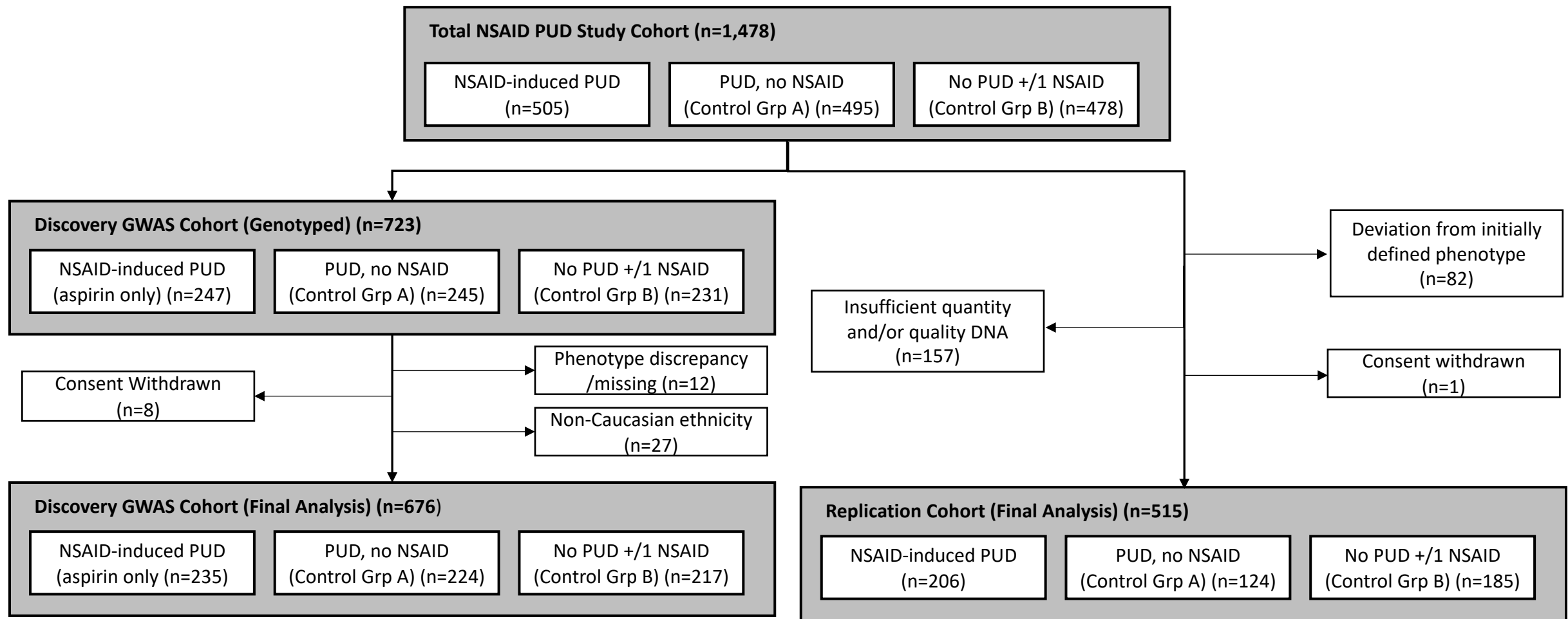

Supplement: Supplementary file 2 [file mmc2.pdf]

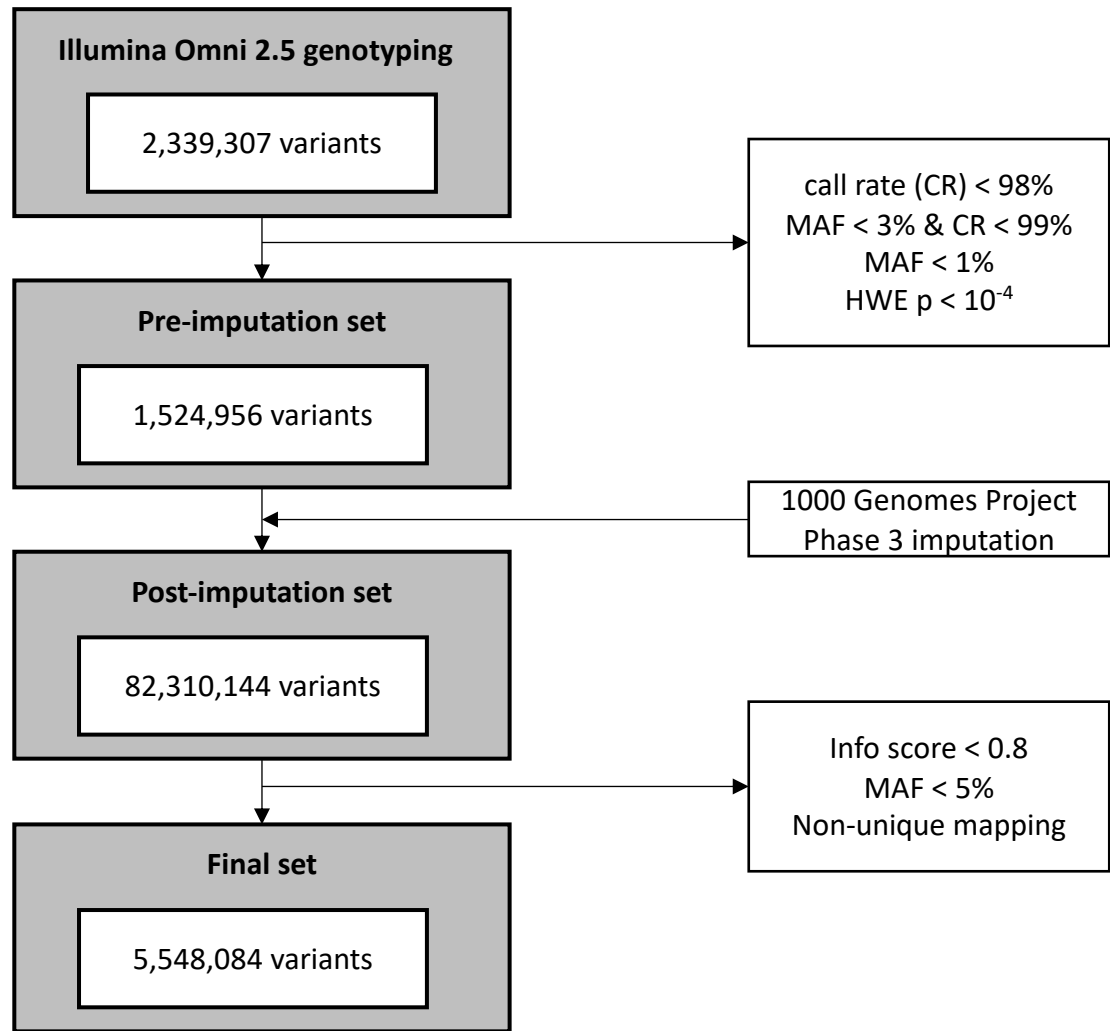

Supplement: Supplementary file 3 [file mmc3.pdf]
