# Supplementary material for: Genome-Wide association between EYA1 and Aspirin-induced peptic ulceration
Source: eBioMedicine. 2021 Dec 2;74:103728. doi: 10.1016/j.ebiom.2021.103728 (PMC8646165; doi:10.1016/j.ebiom.2021.103728)
Supplement: Supplementary file 4 [file mmc4.docx]

**Supplementary Table 1.** Functional impact of variants in LD with rs12678747 as reported by Haploreg v4·1 in Eurasian population from 1000 genome Phase I^1^.

| chr | pos (hg38) | r^2^ | D' | rsID | ref | alt | GENCODE genes | RefSeq genes | dbSNP functional annotation |
| --- | --- | --- | --- | --- | --- | --- | --- | --- | --- |
| 8 | 71195296 | 0·93 | 0·99 | rs730695 | T | C | RP11-326E22·1 | EYA1 | . |
| 8 | 71199364 | 0·97 | 1 | rs10103397 | A | G | RP11-326E22·1 | EYA1 | SYNONYMOUS |
| 8 | 71199443 | 0·97 | 1 | rs10090382 | T | C | RP11-326E22·1 | EYA1 | INTRONIC |
| 8 | 71199504 | 0·98 | 1 | rs10103852 | G | C | RP11-326E22·1 | EYA1 | INTRONIC |
| 8 | 71199989 | 0·96 | 0·98 | rs10104263 | C | T | RP11-326E22·1 | EYA1 | INTRONIC |
| 8 | 71200914 | 0·98 | 1 | rs900109 | A | G | RP11-326E22·1 | EYA1 | INTRONIC |
| 8 | 71200936 | 0·98 | 1 | rs875380 | A | G | RP11-326E22·1 | EYA1 | INTRONIC |
| 8 | 71202049 | 0·96 | 1 | rs4738114 | C | T | RP11-326E22·1 | EYA1 | INTRONIC |
| 8 | 71202541 | 1 | 1 | rs12678747 | A | T | RP11-326E22·1 | EYA1 | INTRONIC |
| 8 | 71203992 | 0·95 | 0·99 | rs6472568 | T | C | RP11-326E22·1 | EYA1 | INTRONIC |
| 8 | 71204667 | 0·96 | 0·99 | rs2380713 | A | G | EYA1 | EYA1 | INTRONIC |
| 8 | 71204731 | 0·96 | 0·99 | rs2380714 | T | C | EYA1 | EYA1 | INTRONIC |
| 8 | 71205109 | 0·94 | 0·97 | rs7016766 | G | A | EYA1 | EYA1 | INTRONIC |
| 8 | 71205170 | 0·96 | 0·99 | rs6472569 | C | A | EYA1 | EYA1 | INTRONIC |
| 8 | 71205270 | 0·95 | 0·99 | rs6472570 | A | G | EYA1 | EYA1 | INTRONIC |
| 8 | 71206912 | 0·94 | 0·97 | rs7828754 | C | T | EYA1 | EYA1 | INTRONIC |
| 8 | 71206935 | 0·94 | 0·97 | rs10539572 | TTA | T | EYA1 | EYA1 | INTRONIC |
| 8 | 71211978 | 0·94 | 0·98 | rs4738116 | C | T | EYA1 | EYA1 | INTRONIC |
| 8 | 71212548 | 0·96 | 0·99 | rs4612370 | A | C | EYA1 | EYA1 | INTRONIC |
| 8 | 71213066 | 0·96 | 0·99 | rs11993577 | T | C | EYA1 | EYA1 | INTRONIC |
| 8 | 71213214 | 0·97 | 0·99 | rs11785838 | A | G | EYA1 | EYA1 | INTRONIC |
| 8 | 71214404 | 0·94 | 0·98 | rs9298164 | C | T | EYA1 | EYA1 | INTRONIC |
| 8 | 71215044 | 0·91 | 0·96 | rs4738117 | C | T | EYA1 | EYA1 | INTRONIC |
| 8 | 71215328 | 0·93 | 0·98 | rs7846086 | G | A | EYA1 | EYA1 | INTRONIC |
| 8 | 71215529 | 0·94 | 0·98 | rs3735935 | C | A | EYA1 | EYA1 | INTRONIC |
| 8 | 71216242 | 0·94 | 0·98 | rs7834524 | T | A | EYA1 | EYA1 | INTRONIC |
| 8 | 71216388 | 0·87 | 0·97 | rs139399702 | G | GGCTAATTCTTCGAAGGAT | EYA1 | EYA1 | INTRONIC |
| 8 | 71216639 | 0·94 | 0·98 | rs4737312 | G | A | EYA1 | EYA1 | INTRONIC |

**Supplementary References**

1. Ward LD, Kellis M. HaploReg: a resource for exploring chromatin states, conservation, and regulatory motif alterations within sets of genetically linked variants. Nucleic Acids Res. 2012;40(Database issue):D930-4.
